# A Personal Perspective on the Initial Federal Health-Based Regulation to Remove Lead from Gasoline

**DOI:** 10.1289/ehp.0800534

**Published:** 2009-04-15

**Authors:** Kenneth Bridbord, David Hanson

**Affiliations:** 1 Division of International Training and Research, Fogarty International Center, National Institutes of Health, Department of Health and Human Services, Bethesda, Maryland, USA; 2 Economic Development Department, Matanuska-Susitna Borough, Palmer, Alaska, USA

**Keywords:** gasoline, government, health-based regulation, lead, policy, U.S. EPA

## Abstract

**Objective:**

This article describes the personal experience and perspective of the authors, who had primary responsibility for drafting the initial health-based regulation limiting lead content of gasoline during the early 1970s while employed by the U.S. Environmental Protection Agency (EPA).

**Data source:**

Information used by the U.S. EPA in developing the initial health-based regulation limiting lead content of gasoline in December 1973 and studies documenting the impact of that and subsequent actions.

**Data extraction:**

Among the lessons learned from this experience is the importance of having input from independent scientists to the regulatory decision-making process. This also demonstrates the critical role of independent peer-reviewed research, such as that supported by the National Institutes of Health, as well as research conducted by scientists from the Centers for Disease Control and Prevention, in delineating the consequences of lead exposure in the population.

**Data synthesis:**

Removal of lead from gasoline in the United States has been described as one of the great public health achievements of the 20th century, but it almost did not happen. The experience of the authors in developing this regulation may be helpful to others involved in developing health-based regulatory policy in the future.

**Conclusion:**

The initial U.S. EPA health-based regulation to remove lead from gasoline is clearly an example where science successfully affected public policy. The leadership of the U.S. EPA at that time deserves much credit for establishing an atmosphere in which this was possible.

Removal of lead from gasoline in the United States has been described as one of the great public health achievements of the 20th century (Needleman 1998a), but it almost did not happen. That it did is a tribute to the hard work, tenacity, and independence of a critical few scientists and government officials in the early 1970s. These scientists and health officials faced enormous opposition but never lost sight of the mandate to protect public health.

The initial health-based regulation to reduce lead content in gasoline was promulgated by the U.S. Environmental Protection Agency (EPA) in 1973 (U.S. EPA 1973d). This was the first regulatory step in a series of actions that dramatically reduced use of leaded gasoline and subsequently blood lead levels across the world. The dramatic impact of the reduction and eventual elimination of lead in gasoline is illustrated by the 98% reduction in the percentage of U.S. children 1–5 years of age with elevated blood lead levels (> 10 μg/dL) from the period 1976–1980 to the period 1999–2002 [Centers for Disease Control and Prevention (CDC) 2005].

We, the authors, had the opportunity and privilege to play major roles in developing the rationale for and drafting the initial federal health-based regulation to remove lead from gasoline. This took place while we were both employed by the U.S. EPA during 1971–1975.

## Background

In the early 1970s, 200,000 tons of lead was emitted from automobiles in the United States each year, mostly in urban areas. Lead was added to gasoline to reduce engine knock in high-compression engines, which otherwise would have required higher-octane gasoline. The oil and lead industries, including manufacturers of gasoline lead additives, had successfully thwarted government efforts to limit lead in gasoline for 50 years (Needleman 2000).

The oil and lead industries used various strategies to forestall regulation of lead in gasoline. For example, when workers involved in the initial manufacture of gasoline lead additives suffered severe lead poisoning and even deaths, the lead industry blamed the victims for failing to follow good work practices. Another strategy employed by the lead industry was to use their public relations capabilities to advertise the benefits of their products to the general public while casting doubt on the possibility of harm associated with use of these products (Denworth 2008; Michaels 2008; Needleman 2000; Rosner and Markowitz 1985). This was particularly true in the case of airborne lead: The lead industry vigorously claimed that airborne lead was a negligible contributor to population lead exposure and was not a factor in excessive lead exposure in children. The lead industry was able to achieve its influence in large part by being the primary supporter of research on health effects of lead and relying upon the scientists that it supported to communicate and interpret this research to the government and the public (Denworth 2008).

As a result, the lead industry was in a position to impede the free flow of scientific information related to the hazards of lead in gasoline, including restrictions on the ability to publish this information without prior approval (Denworth 2008; Rosner and Markowitz 1985). Consequently, the vast majority of relevant studies of lead in gasoline published until the early 1970s were favorable to the lead industries.

To better understand the circumstances of the early 1970s, it is important to recognize the evolution of environmentalism. The decade of the 1960s saw the birth of the environmental movement in the United States, partly in response to the 1962 publication of Rachel Carson’s book *Silent Spring* (Carson 1962). This resulted in actions by Congress to create new federal agencies around 1970 in response to these concerns. These agencies included the U.S. EPA, as well as the National Institute of Environmental Health Sciences (NIEHS) within the National Institutes of Health (NIH), and expansion of the mission of CDC beyond its historic role of protecting the public from communicable diseases. Without the creation of these agencies and passage of the Clean Air Act (Clean Air Act 1970), attributable to Senator Edmund Muskie, it is unlikely that the federal government would have been able to regulate lead in gasoline. Before 1970, there was no federal agency with the clear mandate and authority to protect public health from environmental hazards.

Creation of these new agencies and their expanded environmental health protection mandates required that basic process questions be answered and new regulatory procedures established. Although a huge new area of potential regulatory activity had been created, few people were available in 1971 to address these new fields. The U.S. EPA was in a startup phase similar to the garage workshop phase in the development of desktop computers.

## Our Involvement in the Lead Standard

The story of how we both became involved in the issue of lead in gasoline within the U.S. EPA has many similarities. We both were newly hired U.S. EPA employees in the summer of 1971, David Hanson at the Office of Air Programs in Durham, North Carolina, and Kenneth Bridbord at the National Environmental Research Center in Research Triangle Park, North Carolina. Hanson was just out of graduate school and into his first full-time job. The same was true for Bridbord, who had just completed his master of public health degree after graduation from medical school and an internship in pediatrics.

In late 1971/early 1972, we were both transferred to the headquarters of the U.S. EPA in Washington, DC. We were young, idealistic employees, naive to the ways of Washington and not fully understanding the challenges and difficulties of the responsibilities that we were soon to inherit. We were given these responsibilities mainly because no one else was available to address them.

The regulations and supporting documentation that we both worked on were part of a series of proposals and actions taken by the U.S. EPA in 1972–1973 and the subsequent legal challenges and decisions from 1974 through March 1976.

Late in 1971, Hanson was asked to work with other U.S. EPA employees to develop a rationale and proposal for regulating lead in gasoline. He was moved to the headquarters of the U.S. EPA in Washington, DC, to more efficiently complete this assignment.

Bridbord’s involvement in the issue of lead in gasoline was accidental. The agency needed a physician to participate in the press conference announcing the proposal to regulate lead in gasoline in February 1972. Bridbord was called in because the most senior U.S. EPA health official was not available on that occasion. Despite youth and inexperience, Bridbord inherited primary responsibility for the U.S. EPA assessment of health issues related to lead in gasoline, and Hanson was assigned primary responsibility for the overall regulation, of which health was only one part.

When we began our work on the regulations, there were two major issues related to lead in gasoline. First, lead inactivated catalytic converters. These devices had become the technology of choice for new automobiles to achieve U.S. EPA air pollution standards for carbon monoxide and photochemical oxidants. This required a separate U.S. EPA regulation to provide essentially lead-free gasoline (no more than 0.05 g/gal, vs. “up to” 4 g/gal in leaded gasoline) for all new automobiles with catalytic converters (U.S. EPA 1973a). Second, there was a health basis to reduce lead in gasoline for existing automobiles to accelerate reduction of air lead levels beyond that achievable by lengthy phase-out of older vehicles using leaded gasoline.

In the early 1970s, we experienced considerable resistance to removal of lead from gasoline, not only by industry but also by government and public health scientists. Many scientists asserted that lead in gasoline caused no health effects and referred to a large number of studies supporting that position. Concerns were also raised about the adverse impact the regulation would have on companies that manufactured lead additives and on the oil company refineries. It was further postulated that removing lead would cause gas prices to skyrocket.

These concerns were being voiced against the backdrop of the 1973 gasoline shortage caused by the Arab oil embargo. Cars lined up for blocks waiting to gas up, and in some parts of the country one could only go to the gas station on an even- or an odd-numbered day. The argument was made that the lead regulations would exacerbate the oil shortage by requiring more oil to replace the octane lost by removing lead. With the gas shortage dominating the news, we were informed by certain senior executives that removal of lead from gasoline was politically impossible. Consequently, it was argued, if eliminating lead emissions was necessary, this could be achieved by retrofitting lead traps on automobiles. This alternative turned out not to be feasible for legal and technical reasons (U.S. EPA 1973d).

Resistance to removal of lead from gasoline also resulted in a series of efforts to discredit or influence us. In one instance, industry representatives held meetings with the U.S. EPA administrator in an attempt to discredit Bridbord. Another time, Bridbord was required to provide 2 days of sworn testimony as part of a civil action brought by the major manufacturer of gasoline lead additives against the U.S. EPA in Richmond, Virginia. This discovery deposition was designed to uncover information to be used by the lead industry in future legal action against the agency and to call into question the qualifications of Bridbord. In another incident early in 1972, a lobbyist for a major manufacturer of lead additives offered Hanson employment at a considerable increase in salary. In an effort to convince the U.S. EPA not to issue the health-based regulation, the industry even sent a delegation to try to convince the U.S. EPA administrator that the lead regulation was not necessary because they alleged lead was an essential mineral required for optimum growth and development.

Our youth was also used against us. Our inexperience was cited as a reason for rejecting the lead regulatory proposals at public hearings, during congressional testimonies, and then again during subsequent legal challenges. At one congressional hearing, Hanson was specifically requested by a Texas congressman to state his age for the record.

Finally, even more attention was focused on this issue because this was the first major regulation that the newly formed U.S. EPA moved forward on a nationwide basis.

## Regulatory Proposals and Public Hearings

The U.S. EPA originally proposed a health-based regulation for lead in gasoline on 23 February 1972 (U.S. EPA 1972b). Hanson coordinated this regulatory effort and wrote the proposed regulation. The regulation was based on the U.S. EPA’s interpretation of the Clean Air Act that it did not require irrefutable evidence of harm to take prudent measures to protect the public against widespread lead exposure from automotive emissions.

This proposed regulation for lead in gasoline was based upon the document “Health Hazards of Lead” principally authored by Carl Shy, a senior physician scientist at the U.S. EPA in North Carolina (U.S. EPA 1972a, 1972b). Because this proposal went against conventional wisdom, Hanson had difficulty finding scientists willing to publicly support a health-based regulation. Shy was one of the first physicians to come forward and publicly support regulation of lead in gasoline even though at this time there were few published studies to back him up.

The “Health Hazards of Lead” document addressed the adverse physiologic effects of lead on heme synthesis, particularly at blood lead levels > 40 μg/dL. Based upon this document, the U.S. EPA concluded that these changes were early events leading to more serious damage to the nervous system that was known to occur at higher blood lead levels, and that air lead levels contributed to high blood lead levels. The documents supporting the proposal relied upon the Goldsmith-Hexter regression equation that related blood leads to air lead levels (Goldsmith and Hexter 1967). Based upon this information, the U.S. EPA concluded that air lead levels > 2 μg/m^3^, which were common in many urban areas, were a contributing factor in the number of persons with blood leads exceeding 40 μg/dL. Automobile exhausts were the predominant contributor to these elevated air lead levels in urban areas. As a result, U.S. EPA proposed a regulation to keep air lead below this level by limiting lead in gasoline (U.S. EPA 1972b).

One argument against the U.S. EPA health-based regulation was that there might be no need for a health-based standard because catalytic converters, required to meet automotive emission standards, necessitated use of lead-free gasoline in any case. Consequently, as older vehicles would be replaced with newer ones using catalytic technology, automobile lead emissions would decrease and ambient lead-levels would gradually decline. Fortunately, the need for a health-based standard prevailed. Not only was there a need for a health-based regulation to accelerate removal of lead from gasoline beyond that achievable by phase-out of older vehicles, but also as an insurance policy to guard against introduction of new auto motive technology that could operate with leaded gasoline while achieving the other emission standards. Importantly, establishing a health rationale to remove lead from gasoline in the United States also served as an impetus for other nations to take similar actions. This was significant because globally not all automobiles are required to use catalytic converters.

Public hearings on the initial February 1972 proposed regulations were organized by Hanson. The hearings resulted in many comments on the impact of the proposed regulations on the oil and gas industry and lead industry. These two industries accounted for most of the testimony at the hearings. They testified to the substantial damage they believed the regulation would do to their industries, including lost profits, the inability to fund exploration and development, and difficulties in providing affordable gasoline to the public. Another concern was that removal of lead would endanger the survival of as many as 50 refineries across the country. In fact, only one small older refinery was closed purportedly because of the regulations on lead in gasoline.

The health basis for the regulation was also aggressively attacked during the public hearings. Arguments against the proposed regulation included concern that replacing lead in gasoline with cancer-causing aromatics would be even more harmful and that there was no documented relationship between blood lead in children and lead in gasoline. The industry pressed its viewpoint that air lead was a negligible contributor to lead exposure in the population and that the U.S. EPA had no evidence to support its position that it was.

As a result, the U.S. EPA reexamined the evidence and almost a year later, on 10 January 1973, reproposed a modified health-based regulation (U.S. EPA 1973b). This regulation was supported by a new health document, “EPA’s Position on the Health Effects of Airborne Lead” (U.S. EPA 1972d), principally authored by Bridbord. This document acknowledged limitations in existing data on the air lead–blood lead relationship but also summarized the emerging scientific data on risks to children from exposure to lead in dirt and dust. The document provided evidence that lead in dirt and dust was related to distance from roadways and consequently to use of lead in gasoline.

The revised proposal was designed to limit overall emissions of lead from motor vehicles without tying this reduction to achieving a specific air lead level, because of the limited data available at that time on the air lead–blood lead relationship. The health rationale for the reproposed U.S. EPA regulation was based on the concept that reducing lead in gasoline would reduce the overall lead burden in the United States, particularly for children. The rationale for this action was also described in a presentation at a major international conference on lead held in Amsterdam in the fall of 1972 (Greenfield et al. 1973). During 1973, the U.S. EPA continued to gather additional information with the intent of moving forward with a health-based standard.

The time frame for developing the final regulation was accelerated when the Natural Resources Defense Council (NRDC) filed a motion in the U.S. Court of Appeals for the U.S. EPA to make a decision on the health-based regulation. As a result, the U.S. Court of Appeals on 28 October 1973 ordered the U.S. EPA to make a determination within 30 days whether to regulate lead in gasoline for health reasons (NRDC *v.*U.S. EPA 1973). We both remembered how pleased we were when this motion, filed by David Schoenbrod of NRDC, was accepted by the courts. This increased our optimism that the U.S. EPA would now move forward with this regulation. We both were asked to continue our work on the lead regulations and to lead the effort responding to the court order.

As a result, the U.S. EPA promulgated the initial health-based regulation on 6 December 1973, concluding that automobile lead emissions endanger public health (U.S. EPA 1973d) based upon a third health document, “EPA’s Position on the Health Implications of Airborne Lead” (U.S. EPA 1973c). Bridbord took day-to-day responsibility for coordinating preparation of the third health document, working with a more experienced U.S. EPA health scientist, John Buckley. The final rule making was informed by additional health-related information indicating a correlation between lead in dirt and dust and blood lead levels in children. Appendix 1 summarizes the provisions of this regulation, designed to achieve a 60–65% reduction in lead emissions nationwide by 1980 compared with the 1971 base period. The rationale for this regulation was also described in congressional testimony before then Senator Joe Biden (Bridbord 1974; Quarles 1974).

## Health Rationale

In the 1950s and 1960s, the clinical presentation of lead poisoning in children was associated primarily with lead paint exposure, and major efforts were being made to address the lead paint exposure problem during the 1960s and 1970s. Lead poisoning in children was characterized by overt signs and symptoms, including acute and chronic encephalopathy, peripheral neuropathy, nephropathy, anemia, abdominal pain, and X-ray evidence of lead-containing paint chips in the abdomen. These conditions were associated with blood lead levels in the 60–80 μg/dL range or higher. At higher blood lead levels, severe mental retardation and even death were known to occur. Although there was concern that less severe but still significant effects were occurring at lower blood lead levels, the evidence for lower level effects at that time was not well established. Consequently, there was skepticism in the public health community about efforts to limit lead exposure from gasoline when the problem appeared to be primarily exposure to lead paint in deteriorating housing. There was, in fact, considerable concern that efforts to address lead in gasoline would draw attention and resources away from efforts to address the lead paint problem.

In the early 1970s, the prevailing belief continued to be that the major concern with lead was exposure of children to lead-based paint. During this period, however, there was also increasing evidence of a more generalized contamination of the environment caused by lead emissions from motor vehicles. This was characterized not only by ambient air lead levels but also by high levels of lead in dirt and dust measured in the thousands of parts per million levels. Although the most severe cases of lead overexposure were found among children living in housing with deteriorating lead paint, there also was a more widespread blood lead elevation in the general population, particularly in children living close to heavily trafficked urban roads but not necessarily living in deteriorating housing (U.S. EPA 1973d). This represented a second epidemic caused by lead in gasoline, in addition to the epidemic caused by lead paint.

The U.S. EPA decided not to link the final regulation to the originally proposed air lead level of 2 μg/m^3^. This was because the existing air lead–blood lead correlation studies did not control for the multiple sources of lead exposure in the general population and did not account for lead in dirt and dust, which was an important source of exposure for children. In modifying its position, the U.S. EPA considered airborne lead to be an important contributor to lead body burden in both children and adults, and especially in children, through ingestion of dirt and dust contaminated by lead from motor vehicle exhausts.

It is noteworthy that the U.S. EPA subsequently did establish a national ambient air quality standard for lead of 1.5 μg/m^3^ in 1978 and later revised this standard to 0.15 μg/m^3^ in 2008 (Needleman 2000; U.S. EPA 2008). Except for the need to better control lead emissions from stationary sources, the one action that has generally made it possible to meet the original as well as the revised air quality standard for lead was the phased reduction and ultimate elimination of leaded gasoline in the United States.

## Interpretation and Use of Scientific Information

During the period when the U.S. EPA was developing its approach to lead in gasoline, the agency had contracted with the National Academy of Sciences, National Research Council (NRC) for what was anticipated to be an independent scientific assessment of airborne lead (NRC 1972). However, there was concern that the NRC report did not provide an independent analysis, because a number of panel members, consultants, and contributors were affiliated with or had been supported by the oil and lead additive industries (Gillette 1971). Consequently, the report, which was released in 1972, was not relied on by the U.S. EPA to form the basis for the agency’s health assessment of lead in gasoline. In contrast, the NRC report was used by the lead industry in public relations efforts to say there was no health concern with lead in gasoline (Denworth 2008; Needleman 2000).

One of the scientists who first called attention to the risks posed by lead in gasoline was Clair Patterson, a highly respected geochemist at the California Institute of Technology. Patterson was among the most prominent scientists to raise concern about the need to reduce lead emissions from gasoline. Research conducted by Patterson documented the great buildup of lead in the environment and in people because of industrial activity in general, and combustion of gasoline containing lead additives in particular (Denworth 2008; Needleman 1998b, 2000; Patterson 1965). The U.S. EPA considered the likelihood that the studies and conclusions of Patterson were in fact correct, which ultimately they were shown to be (Denworth 2008). The main reason that the U.S. EPA did not rely on Patterson’s studies to justify its health basis for removal of lead from gasoline was the difficulty in relating these findings in a quantitative way to specific levels of lead use in gasoline. If the U.S. EPA had used the Patterson studies, they would have been seen as a major rationale for the regulation. Given the considerable high-profile effort that the lead industry had expended to challenge the studies of Patterson, there was concern that if the Patterson studies were successfully challenged in any future legal proceeding, this could jeopardize the health-based regulation. As a result, the U.S. EPA decided to sidestep the debate over interpretation of the Patterson studies and to base its health rationale on a broad body of other emerging scientific information.

Studies by Philip Landrigan and colleagues at the CDC were particularly informative to U.S. EPA in documenting the hazards from exposure to elevated levels of lead in dirt around stationary lead sources, in particular, the El Paso lead smelter (CDC 1973; Landrigan et al. 1975). This study was one of the first to demonstrate a relationship between soil and household dust lead levels and blood lead levels in children and young adults. Newer studies also provided more documentation of high levels of lead in dirt and dust near heavily traveled roadways (U.S. EPA 1973c, 1973d).

Subsequent studies by Herbert Needleman and colleagues documented the adverse consequences of low-level lead exposure in children on intelligence and behavior (Needleman et al. 1979). Further studies have confirmed the correctness of the conclusions of Needleman’s research on the effects of lead on intelligence and behavior (Denworth 2008). These studies were based on independent peer-reviewed research supported by the NIH and especially by the NIEHS, as well as the National Institute of Child Health and Human Development (NICHD). As a result, the scientific community has been better able to understand the health risks from exposure to lead (Canfield et al. 2003; Cecil et al. 2008; Gilbert and Weiss 2006; Jusko et al. 2008; Lanphear et al. 2000, 2005; Needleman et al. 1979, 1990; Wright et al. 2008).

## The Scientific Community and the Seven Questions

The experience regarding lead in gasoline reinforces how vitally important independent input from the medical, public health, and scientific community is to a federal agency developing regulatory policy. Before the U.S. EPA paper “Health Effects of Airborne Lead,” released in late 1972, an announcement was placed in the *Federal Register* and widely circulated in the scientific community requesting responses to seven questions (U.S. EPA 1972c), which were formulated by Bridbord. An example of one of these questions was: How much of a hazard is dust-fall lead for children? Appendix 2 lists all seven questions.

The *Federal Register* notice sent by acting U.S. EPA administrator Robert Fri both extended the comment period on the February 1972 health-based proposal and sought wide input from the scientific community and other government agencies responding to the seven questions. This action reflected the U.S. EPA’s determination that it had a responsibility to reach out to the scientific community to seek the most recent information related to the health hazards of lead under the new Clean Air Act authority to protect public health. Responses received provided considerable new scientific information.

Comments received pointed out limitations of the existing data on the relationship between air lead and blood lead, including the Goldsmith-Hexter regression equation (Goldsmith and Hexter 1967). In place of the relationship between air lead and blood lead levels, emphasis was now placed on hazards to young children from exposure to lead-contaminated dirt and dust resulting from automotive lead emissions.

Responses to the seven questions not only provided invaluable scientific information, but also helped to mobilize additional research. That research was discussed at a major conference on low-level lead toxicity convened by U.S. EPA and NIEHS in North Carolina in October 1973 and subsequently published in *Environmental Health Perspectives* (Low-Level Lead Toxicity 1974). Several of the papers from the U.S. EPA/NIEHS conference were ultimately cited in the third U.S. EPA health document (U.S. EPA 1973c), which was used as the health basis for the final regulations.

## Reactions of Other Federal Agencies

The U.S. EPA regulations on lead in gasoline were proposed shortly after a major reorganization in which employees from a number of federal agencies were reassigned to staff the newly created U.S. EPA. Most U.S. EPA staff came from the U.S. Public Health Service (PHS), part of the Department of Health, Education, and Welfare (DHEW). As a result of this reorganization, many U.S. PHS staff retired before or shortly after being transferred to the U.S. EPA, leaving a vacuum at the U.S. EPA that was filled by younger people such as ourselves. This created jealousies and unhappiness among remaining U.S. PHS and U.S. EPA staff. This was one of the reasons that DHEW, the department that traditionally addressed air pollution, was so actively opposed to a health-based regulation.

In addition, DHEW initially questioned whether the U.S. EPA even had the authority to regulate lead in gasoline. The fact that DHEW had to share health protection responsibilities with the U.S. EPA may be one of the reasons for the consistently strong negative views on this regulation taken by officials in the Office of the Assistant Secretary for Health. On one occasion, we met with the assistant secretary for health, who was skeptical about the need for this regulation. U.S. EPA administrator William Ruckelshaus sent DHEW secretary Elliot Richardson a letter requesting comments on the above-mentioned seven questions. The responses were not very supportive. In a communication to Senator John Tunney, Richardson was especially critical of the U.S. EPA taking action to remove lead from gasoline based upon health considerations, stating that “there is no firm evidence at this time that lead derived from combusted gasoline is harmful to the health of the general public” (Richardson 1973). However, the communication to Senator Tunney acknowledged that the U.S. EPA now had the statutory authority to take this action.

Ruckelshaus and Richardson, based upon advice from their respective staff, had differing views about the need for a health-based standard. Richardson and Ruckelshaus subsequently became the U.S. Attorney General and U.S. Deputy Attorney General, respectively, who were later fired by President Nixon for failing to dismiss the Watergate prosecutor. They obviously had great respect for each other even though they had different perspectives with regard to lead in gasoline.

Industrial opponents to removal of lead from gasoline were very adept at taking advantage of the differing views related to the health impact of lead in gasoline and cultivating support from scientists most concerned with the problem of lead in paint. This was also one of the reasons why leadership at DHEW disagreed with and actively opposed the health rationale used by the U.S. EPA to remove lead from gasoline. DHEW and other federal agencies, such as the Department of Transportation and Department of Commerce, vigorously opposed a health-based regulation at the level of the Office of Management and Budget (OMB).

An important person at OMB who, after considering all of the evidence, agreed with the U.S. EPA position was John Sawhill, deputy director of the OMB. If it were not for Sawhill’s support, the U.S. EPA health-based regulation for lead in gasoline would have stalled and might never have been promulgated. Sawhill was able to support the U.S. EPA position because of the very thorough research and analyses that had been completed by U.S. EPA staff addressing the criticisms and questions raised about the proposed regulations. This information enabled us to answer and rebut all questions and charges lodged by representatives of the other federal agencies that were against the regulation. Sawhill later became director of the Nature Conservancy.

As a reflection of the evolving views of the scientific community, the DHEW Committee to Coordinate Toxicology and Related Programs in 1975 requested that a departmental committee be formed to assess the human health consequences of lead exposure from automobile emissions. The results of this effort were published in *Environmental Health Perspectives* (Falk 1977). The committee recommended “phasing-out of lead in gasoline by reducing its content as has been proposed in the U.S. by EPA and by many other developed countries.”

## Legal Challenges to the Standard

After promulgation of the 1973 U.S. EPA health-based lead in gasoline regulation, there were a number of legal challenges, which included attacks on the authors’ background and qualifications. The initial challenge by the lead industry, heard by three justices in the U.S. Court of Appeals on 9 September 1974, resulted in this regulation being set aside on 28 January 1975 (Ethyl Corp. *v.* U.S. EPA 1974). A subsequent appeal heard en banc (the entire Court of Appeals panel of the U.S. Court of Appeals in Washington, DC) on 30 May 1975 resulted in a decision to affirm the regulation on 19 March 1976 (Ethyl Corp. *v.* U.S. EPA 1976). The Supreme Court ultimately did not agree to hear an appeal of this decision, in effect upholding the determination of the lower court, allowing the lead phase-down regulation to take effect in 1976.

The ability of the U.S. EPA to prevail in these legal challenges was attributed both to the care that had been taken in developing the final regulation and to the dedication and competence of U.S. EPA attorneys such as Leslie Carothers who presented and defended the regulation in subsequent legal challenges. The U.S. EPA also benefited by having competent officials such as Rick Penna, who was responsible for developing the enforcement strategies for the final regulation. The authors also very much appreciated the competent support provided by another U.S. EPA attorney, Gerry Gleason, who represented us during various depositions and other legal challenges by the lead industries.

## Observations

**Figure 1:**
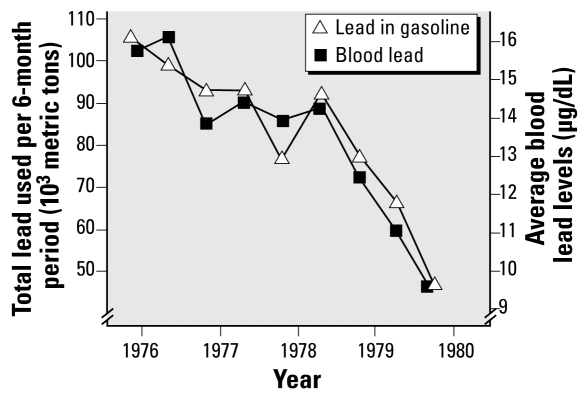
Parallel decreases in blood lead values and amounts of lead consumed in gasoline between 1976 and 1980 (U.S. EPA 1986).

Removal of lead from gasoline has been particularly beneficial for the health of children in the United States. In 2005, the CDC documented a 98% reduction in the percentage of U.S. children 1–5 years of age with elevated blood lead levels (> 10 μg/dL) from the period 1976–1980 to the period 1999–2002 (CDC 2005). Evidence of this decline began to appear in the 1980s (Annest et al. 1983; CDC 1997; Needleman 2000; Pirkle et al. 1994). Although reduction of lead exposure from a number of sources, particularly food and paint, contributed to this decrease, the action believed most responsible for this rapid and dramatic decline in blood lead levels was removal of lead from gasoline (Annest et al. 1983; Needleman 2000; Pirkle et al. 1994). This conclusion is supported by the close temporal relationship between decreased use of lead in gasoline and subsequent rapid declines in blood lead levels both in the United States and in other countries, as illustrated in Figure 1. For example, studies on six continents have documented an extremely strong correlation between reductions in blood lead and decreased use of lead in gasoline (Thomas et al. 1999).

The regulation that we both worked on represented the first successful effort by the federal government to regulate lead in gasoline since questions were raised about this 50 years earlier (Needleman 2000). Removal of lead from gasoline can be considered the environmental health equivalent of removing the handle from London’s Broad Street Pump, whose contaminated water was the source of the 1854 cholera epidemic in London.

As blood lead levels in children have decreased, the blood lead level of concern for children has also decreased from 40 μg/dL in the early 1970s to the current level of 10 μg/dL. It would not have been possible to identify these lower level effects until after the gasoline lead phase-out had begun. This is because before this time, virtually all children in the U.S. had blood levels exceeding the 10-μg/dL level. With continuing research, questions have been raised regarding whether blood leads even at or below 10 μg/dL are harmful to children (Canfield et al. 2003; Jusko et al. 2008; Lanphear et al. 2000, 2005; Mead 2008; Needleman et al. 1990).

The effort to remove lead from gasoline provides several additional observations. First, the ability of the U.S. EPA to take this regulatory action was greatly facilitated by research conducted by government and independent scientists not supported by industry. This illustrates the crucial importance of independent peer-reviewed research to better understand the health consequences of lead exposure in the population. This support was primarily provided by the NIEHS, NICHD, and CDC.

Second, it takes a long time before new information affects medical and public health practice. It was not until the results of this research began to appear in high-impact scientific journals such as the *New England Journal of Medicine* that the medical and public health communities more fully appreciated the hazards posed by lead, including airborne and dust-fall lead in general and lead in gasoline in particular (Annest et al. 1983; Landrigan et al. 1975; Lin-Fu 1972, 1973a, 1973b; Needleman et al. 1979, 1990).

Third is the importance of basing regulatory decisions on a broad foundation of scientific studies so that legal challenges are not easily able to attack the credibility of a single or a limited number of studies or individuals. This was a consideration in the U.S. EPA not tying the final health-based regulation to a specific air lead level, which would have required relying on the Goldsmith-Hexter regression equation, an approach that had been criticized both during the first public hearing and by independent scientists in response to the seven questions outlined in Appendix 2 (Goldsmith and Hexter 1967; U.S. EPA 1972d). The subsequent unsuccessful attempt by the lead industries to discredit the findings of Herbert Needleman in an effort to call into question the health basis to regulate lead in gasoline in the 1980s and the early 1990s also illustrates this point (Denworth 2008; Needleman 1992; Palca 1992; Rosner and Markowitz 2005; Silbergeld 1995).

Fourth, development of a health-based standard to remove lead from gasoline would not have been possible without the support of the U.S. EPA leadership. This support was essential in providing us the time and opportunity to build the strongest case for the removal of lead from gasoline to protect public health under the Clean Air Act authority. Leadership support was provided by the U.S. EPA administrators, acting administrators, and deputy administrators William Ruckelshaus, Russell Train, Robert Fri, and John Quarles, along with assistant administrators Robert Sansom and Stanley Greenfield. Other U.S. EPA health officials who provided invaluable support in developing the health justification for this action were Vaun Newill, Robert Horton, John Buckley, Carl Shy, and John “Jack” Finklea.

Fifth, in the early 1970s, scientists at U.S. EPA were free to examine and evaluate the growing body of scientific evidence as the basis for the initial health-based regulation. At no time was there ever internal U.S. EPA political pressure to change scientific interpretations or conclusions. The initial U.S. EPA health-based regulation to remove lead from gasoline is clearly an example where science successfully affected public policy. The leadership of the U.S. EPA at that time deserves much credit for establishing an atmosphere in which this was possible.

Sixth is the critical role played by non-governmental organizations, such as the NRDC, in influencing regulatory policies. David Schoenbrod from the NRDC deserves major credit for the role that the NRDC played in legally challenging the U.S. EPA to take regulatory action to remove lead from gasoline.

Seventh, we were given the opportunity to succeed because we were expected to fail. One of the reasons we did not receive more opposition from skeptics within the U.S. EPA was that very few people there believed we could succeed, so they just left us alone. One high-placed U.S. EPA official openly stated that with the lines at the gas pumps and the strong industry opposition, we did not have a chance and that we were merely sacrificial lambs. This was further supported by the fact that Hanson was sent to OMB alone to defend the second proposed lead regulation in front of representatives of the various federal agencies in much higher positions than Hanson. He later learned that he was sent alone, as a relatively low-level agency representative, because many expected this effort to fail.

Finally, in retrospect, our youth and inexperience also helped us to succeed. We were too young to know that regulating lead in gasoline was impossible. Our youthful dedication, hard work, and competent analyses overcame obstacles that were much more formidable than we recognized. The two authors of this paper were recognized for their contributions to the lead regulation at an unusually young age as recipients of Silver Medals for Superior Service by the U.S. EPA, the second highest award given by the agency.

We both consider ourselves privileged to have had the opportunity to contribute to this action, which has had such a beneficial impact in the United States as well as in many other countries.

## Addendum

It is beyond the scope of this article to recognize all of the other persons who over the years have made important contributions to reduction of lead exposure, not only from gasoline but also from other sources, particularly lead in paint and food. A more comprehensive account of the contributions of these individuals can be found in Lydia Denworth’s book *Toxic Truth* (2008).

Members of the task force who contributed to the third U.S. EPA health document, in addition to John Buckley and Kenneth Bridbord, included Douglas Hammer, Robert Horton, Marty Kanarek, Wellington Moore, Magnus Piscator, Lawrence Plumlee, Steven Reznek, Richard Rhoden, and Jerry Stara (U.S. EPA 1973c).

Members of the committee who contributed to the 1975 DHEW report on lead exposure from automobile emissions in addition to Hans Falk included Terri Damstra, Kathryn Mahaffey, Warren Piver, and Herbert Posner (Falk 1977).

The initial lead in gasoline phase-down and subsequent U.S. EPA regulations would not have been possible without significant support from the scientific community. Based upon the personal knowledge of the authors, these individuals include, but are not limited to, scientists such as Herbert Needleman, Sergio Piomelli, and Ellen Silbergeld, as well as health officials at other federal and state agencies, such as Philip Landrigan, Vernon Houk, Edward Baker, and Henry Falk at CDC; Jane Lin-Fu at DHEW; John Goldsmith from the California State Health Department; and David Rall, Director of NIEHS. The three papers published in the *New England Journal of Medicine* by Jane Lin-Fu (1972, 1973a, 1973b) were especially influential in helping the scientific community, including those at the U.S. EPA, to reassess the situation with respect to lead exposure and its implications in the United States.

Regulations once promulgated must be upheld and enforced to do their job. Joel Schwartz, who followed us at the U.S. EPA, deserves major credit for his role in preventing the regulation that we worked on from being undone during the antiregulatory era of the 1980s and for conducting the analyses that ultimately formed the basis for total elimination of lead in gasoline in the United States. These analyses included the data in Figure 1, which show a close relationship between decreases in blood leads and reduction of leaded gasoline use resulting from the initial lead phase-down regulations that we both were responsible for (Annest et al. 1983; U.S. EPA 1986).

Subsequent to our own involvement, both Joel Schwartz and Ellen Silbergeld were recipients of MacArthur Awards for their important work on lead (Denworth 2008; Schmidt 2005). Herbert Needleman received the Heinz Award for the Environment for his major contributions to lead research and regulatory policy (Denworth 2008).

One person who is widely recognized for her contributions in reducing dietary lead exposure is Kathryn Mahaffey while at the U.S. Food and Drug Administration (Denworth 2008). Kathryn Mahaffey is also the person who requested that lead be included in the National Health and Nutrition Examination Survey which made possible documentation of the decreasing trends in blood leads in the U.S. population as shown, for example, in Figure 1 (Denworth 2008; see also Anonymous 2009).

## Appendix 1 Provisions of the 1973 U.S. EPA Health-Based Lead Phase-Down Regulation: Limits on Allowable Levels of Lead in Gasoline

Maximum allowable lead levels in the manufacture of gasoline (U.S. EPA 1973d):

- 1.7g lead/gal, after 1 January 1975
- 1.4 g lead/gal, after 1 January 1976
- 1.0 g lead/gal, after 1 January 1977
- 0.8 g lead/gal, after 1 January 1978
- 0.5 g lead/gal, after 1 January 1979

For each 3-month period (January–March, April–June, July–September, October–December), the average lead content per gallon shall be computed by dividing total grams of lead used at a refinery in the manufacture of gasoline by total gallons of gasoline manufactured at such refinery.

## Appendix 2 Synopsis of the U.S. EPA’s Seven Questions (U.S. EPA 1972c)

- What are the permissible uses and limitations of the Goldsmith-Hexter regression equation for obtaining reasonable estimates of blood lead levels as a function of air lead exposure?
- How accurate a reflection is blood lead of lead body burden, and what is the effect of elevated blood lead upon lead body burden?
- How accurate are estimates used by the U.S. EPA, which were contained in an NRC report, for estimates of daily air inspired by an average adult?
- What is an appropriate safety factor for extrapolating industrial threshold limit values to the general population, and is it appropriate to use these limits for that purpose?
- How much of a hazard is dust-fall lead for children?
- How effective would reductions in other environmental sources of lead exposure, besides lead paint, be in helping to reduce the risk of undue lead exposure among children also exposed to lead paint? How clear is it that all lead poisoning in children is, in fact, caused only by lead paint?
- What is the consequence upon the environment in general of allowing large quantities of lead to be expelled into the atmosphere from motor vehicle exhausts?
